# Wastewater-based surveillance of ESKAPE pathogens in resource-limited countries (RLCs): an essential tool for monitoring antibiotic resistance

**DOI:** 10.1007/s10661-026-15243-y

**Published:** 2026-03-31

**Authors:** Nafyad Ibrahim Batu, Ilunga Kamika, Tshepo Joseph Malefetse

**Affiliations:** Institute for Nanotechnology and Water Sustainability (iNanoWS), College of Science, Engineering and Technology, University of South Africa, Florida Science Campus, Johannesburg, 1709 South Africa

**Keywords:** Antibiotic-resistant bacteria, Antibiotic resistance genes, ESKAPE pathogens, Monitoring, Resource-limited countries, Wastewater-based surveillance

## Abstract

Antibiotic-resistant bacteria (ARB) among ESKAPE pathogens represent one of the most serious public health threats. Wastewater-based surveillance (WBS) has emerged as a cost-effective and noninvasive approach for the early detection of antibiotic resistance genes (ARGs). It holds particular promise in resource-limited countries (RLCs), where traditional clinical surveillance often faces infrastructural and financial constraints. This review examines sampling strategies, pathogen detection technologies and relevant case studies, while identifying barriers such as fragmented sanitation systems, insufficient laboratory resources and the lack of standardised protocols for monitoring ESKAPE pathogens, recognised as critical threats in the WHO Bacterial Priority Pathogens List (BPPL), in RLCs. WBS complements existing surveillance systems, providing early warning of trends and informing timely public health interventions. Furthermore, this review proposes a roadmap for integrating WBS into public health frameworks in RLCs. To maximise its potential, scalable and cost-effective sampling and analysis methods tailored to local infrastructure must be developed, alongside clear ethical and regulatory frameworks to address privacy and data-use concerns. In conclusion, the WBS represents a transformative opportunity for public health monitoring in RLCs. Despite logistical and infrastructural hurdles, its capacity to deliver population-level insights into disease dynamics makes it an invaluable component of a modern surveillance system.

## Introduction

Antibiotic resistance is one of the most serious public health threats of the twenty-first century. Antibiotic-resistant ESKAPE pathogens, a group that includes *Enterococcus faecium*, *Staphylococcus aureus*, *Klebsiella pneumoniae*, *Acinetobacter baumannii*, *Pseudomonas aeruginosa* and Enterobacter species, have increased the burden of disease and contributed to higher death rates **(**Bazira et al., 2025; Ravi & Singh, 2024)*.* These pathogens are responsible for a significant proportion of hospital-acquired infections and are known for their ability to “escape” the effects of commonly used antibiotics. Their multidrug-resistant (MDR) nature not only complicates clinical treatment but also increases the risk of prolonged illness, higher healthcare costs and mortality, especially in resource-limited countries (RLCs) (Seid et al., 2025).

In many RLCs, inadequate health care systems, poor sanitation infrastructure and limited access to laboratory-based diagnostics hinder the timely detection and control of antibiotic resistance threats (Rony et al., 2023)*.* Traditional clinical surveillance methods, which rely on patient-based sampling and diagnostic testing, are often limited by underreporting, high costs and restricted geographic coverage. These challenges underscore the urgent need for alternative, population-level surveillance strategies that can operate effectively within RLCs (Hamilton et al., 2024).

Wastewater-based surveillance (WBS) has emerged as a timely, cost-effective and noninvasive approach for monitoring the presence and spread of antimicrobial-resistant bacteria (ARB) and antibiotic resistance genes (ARGs) in communities (Rahman et al., 2023; Ramos et al., 2024). By analysing the biological content of wastewater, WBS can detect both symptomatic and asymptomatic carriers, offering real-time insights into pathogen spread and resistance trends (Parkins et al., 2024; Singh et al., 2024). This approach is especially valuable for ESKAPE pathogens whose DNA, RNA or viable forms can be identified in wastewater through molecular and culture-based methods. Furthermore, the detection of key ARGs, such as blaKPC, blaNDM, blaimp-1, mecA, blaCTX-M, blaSHV, blaTEM and blaKPC, in sewage reflects the community’s ongoing resistance burden (da Silva et al., 2024; Keer et al., 2025; Zhang et al., 2020).

However, despite its rising global application, the implementation of WBS in RLCs remains limited by context-specific challenges. These include fragmented or informal wastewater networks, limited laboratory infrastructure, inconsistent sampling strategies and the absence of standardised protocols (Cohen et al., 2024; Daza-Torres et al., 2024). On the other hand, several studies from RLCs have demonstrated the feasibility of using WBS to monitor ESKAPE pathogens and their resistance genes, suggesting that, with appropriate adaptation, this tool could help bridge critical surveillance gaps (Rahman et al., 2023; Woodford et al., 2025). Understanding context-specific criteria such as cost-effectiveness, simplicity of sampling, minimal dependence on cold-chain logistics and analytical methods can help to ensure the feasibility and sustainability of implementing WBS in RLCs. Although the WBS approach has been widely studied, only a limited number of studies have examined its applicability in RLCs. In this review, we summarise the key trends in ESKAPE pathogens, the current and potential applications of WBS, adaptation strategies and critical research gaps in surveillance. From this perspective, the aspiration here is to assess existing knowledge and provide an up-to-date framework of monitoring ESKAPE pathogens and an early warning system within RLC contexts.

## Scope and approach of the review

For this review, the application of the WBS of ESKAPE pathogens in RLCs was presented. Among known databases, for retrieving high-quality studies related to the scope of this review paper, the major databases, including Cochrane Library, Google Scholar, Scopus, PubMed and Web of Science, were used. The first screening of the literature was based on the suitability of the title and abstract of the articles, with emphasis on studies that relied on the wastewater-based surveillance. Initially, nearly 370 articles were downloaded, of which only 43 were screened for their suitability and directly stated the application of WBS for monitoring ESKAPE pathogens in RLCs. Due to the studies related to wastewater surveillance in RLCs being limited, this review prioritises a critical analysis and synthesis of existing studies.

## Sources of antibiotic-resistant ESKAPE pathogens and ARGs in wastewater

Wastewater from hospitals and WWTPs’ effluents serves as a complex source for antibiotic-resistant ESKAPE pathogens and their associated resistance genes (Aguilar-Salazar et al., 2023). WWTPs are potentially exposed to ARB and ARGs from the infected individuals through the feces, and to a slight extent through urine, nasal mucus and sputum. Gut pathogenic bacteria such as *Enterobacter* spp., *Enterococcus* spp., *K. pneumoniae*, and *E. coli* are frequently detected ARB in the wastewater (Tiwari et al., 2022). Furthermore, the prevalence of ARB (specifically ESKAPE pathogens) and ARGs in hospital effluents also represents a significant source due to the extensive use of antibiotics (Bakon et al., 2023). In this context, therefore, wastewater is considered to be the principal matrix in preserving and disseminating antibiotic-resistant ESKAPE pathogens and associated resistant genes.

## Key trends in ESKAPE pathogens

### Carbapenemases

Carbapenemases, enzymes that confer resistance to carbapenems, have become increasingly prevalent in Enterobacterales, particularly *K. pneumoniae* and *Enterobacter* species, across various regions, including RLCs. The most common types of carbapenemases include KPC, NDM and OXA-48-like enzymes, each of which contributes to significant challenges in antimicrobial resistance (Alvisi et al., 2025; Lazar et al., 2024). These enzymes have been frequently reported in RLCs. For instance, in Burkina Faso, carbapenemase production was detected in 2.9% of ESBL-producing Enterobacterales, with NDM and OXA-48 being prominent (Garba et al., 2025). Similarly, a study in Thailand identified multiple genetic variants of the blaOXA-48-like gene among carbapenem-resistant Enterobacterales (Santajit et al., 2024).

Both *K. pneumoniae* and *Enterobacter* species are significant contributors to the global problem of MDR infections. They cause infections, including pneumonia, urinary tract infections (UTIs), liver abscesses, meningitis, septicaemia, wound infections and sepsis (Kathi, 2024; Pu et al., 2024). These bacteria exhibit resistance through various mechanisms, including the production of extended-spectrum beta-lactamases (ESBLs), carbapenemases (bla_KPC_, bla_NDM_, bla_OXA-1_, bla_TEM-1b_, bla_SHV-1_ and bla_CTX-M-15_) and biofilm formation (Singh et al., 2025; Wang et al., 2020). *K. pneumoniae* and Enterobacter spp. exhibited high resistance rates to antibiotics, particularly penicillins, cephalosporins and carbapenems, across all regions (Debabza et al., 2018; Kagambèga et al., 2023).

### Multidrug resistance in nonfermenters

*A. baumannii* and *P. aeruginosa* are significant nosocomial pathogens, particularly in intensive care units (Lazar et al., 2024; Venkateswaran et al., 2023). Both species produce β-lactamases, which have a wide diversity of existing enzymes that inactivate antibiotics (Jácome et al., 2016). *A. baumannii* is a Gram-negative, nonmotile, glucose-nonfermentative coccobacillus responsible for a variety of community- and hospital-acquired infections. It is one of the most challenging ESKAPE bacteria involved in adhesion, invasion, cytotoxicity and resistance to carbapenems. *A. baumannii* frequently produces oxacillinase (OXA-type) β-lactamase (carbapenemases), which are the primary mechanisms for carbapenem resistance (Colquhoun et al., 2025). *A. baumannii’s* ability to survive in harsh environments, including hospital settings, is due to its genomic plasticity and various stress response mechanisms (Karampatakis et al., 2024; Lucidi et al., 2024). This is primarily due to various mechanisms such as the production of OXA-type, metallo-β-lactamases, efflux pumps and decreased target access (Khuntayaporn et al., 2021).

*P. aeruginosa* is a common cause of ventilator-associated pneumonia and other nosocomial infections. It exhibits intrinsic resistance, including low outer membrane permeability, efflux pumps, quorum-sensing, and constitutive expression of AmpC β-lactamase to many antibiotics and can acquire additional resistance mechanisms through HGT (Kumar & Vasundhara, 2023; Parasuraman et al., 2020). Ubiquitous circulation in the environment has become a major health threat to humans due to the constant emergence of drug-resistant strains (Liao et al., 2022). *Pseudomonas aeruginosa* often harbours metallo-β-lactamases (MBLs), such as the OprD gene, which affects the entry of carbapenems (Al-Ouqaili, 2018).

### Methicillin-resistant Staphylococcus aureus and vancomycin-resistant enterococci

Methicillin-resistant *Staphylococcus aureus* (MRSA) and vancomycin-resistant enterococci (VRE) remain major contributors to hospital- and community-acquired infections. *S. aureus* is a Gram-positive bacterium capable of causing a spectrum of illnesses ranging from mild skin infections to severe systemic diseases. MRSA is a leading cause of skin, soft tissue, bloodstream and respiratory infections, which are often difficult to treat and control (Joshie & Patil 2023). Its resistance arises through multiple mechanisms, including the production of enzymes that inactivate antibiotics, target site modifications, the action of efflux pumps and plasmid-mediated resistance (Guo et al., 2020). The coa, mecA and spa genes are key mediators of MRSA resistance, underpinning its prevalence in both hospital-associated (HA-MRSA) and community-associated (CA-MRSA) infections (Al-Ruwaili, 2018).

Vancomycin-resistant strains (VRE) are of increasing concern due to their resistance to multiple antibiotics and their capacity to survive under harsh environmental conditions (Geraldes et al., 2022). Key risk factors for VRE infection include prolonged hospitalisation, prior antibiotic exposure and immunocompromised states such as HIV/AIDS (Regasa Dadi et al., 2021; Zike et al., 2024). These infections are associated with high morbidity and mortality (Wagner et al., 2025). VRE strains, particularly those harbouring vanA/vanB, are increasingly reported in hospital settings across multiple RLCs, further complicating the management of bloodstream and device-associated infections (Iqbal et al., 2024).

## Methodological approaches for monitoring ESKAPE pathogens in wastewater

### Sampling techniques

WBS offers a cost-effective and practical alternative for early pathogen detection in settings where conventional disease surveillance is limited (Mao et al., 2020). The selection of an appropriate sampling method, whether grab, composite or passive, should be guided by factors such as local infrastructure, population size and specific public health priorities (Wilson et al., 2022).

#### Grab sampling

Grab sampling is a traditional method, but routine implementation is complex. A sample is collected at a specific time point to assess the presence of a target pathogen in a system. Although this approach is straightforward in concept, it can easily miss periods of peak shedding because it captures only a brief snapshot (Hawker et al., 2022). Consequently, it may fail to represent the true variability of the target over time. Due to the limited spatial representation, traditional grab sampling may poorly indicate public health risks, as it captures data from a single location at a single time point (Bata et al., 2025). Moreover, routine grab sampling is labour-intensive and expensive, with significant salary costs, especially when frequent sampling is required to capture temporal variations (Chapin, 2015).

#### Composite sampling

Composite sampling involves collecting a higher fraction of the discrete samples over a period of time and combining them with a single representative sample (Cha et al., 2023). This sampling technique provides a more accurate representation of microbial loads and consistently gives a diverse collection of microbes (Huijbers et al., 2023). Composite samples can be collected using time-proportional, volume-proportional and flow-proportional techniques, depending on sampling objectives and system design (Verlicchi & Ghirardini, 2019). However, the requirement for automated samplers, continuous power supply and refrigerated storage can limit its application in RLCs. Composite sample collection is, therefore, more costly, time-consuming, increases the risk of contamination, and is labor-intensive, and it may not be feasible under certain circumstances in RLCs (Liu et al., 2022).

#### Passive sampling

Passive sampling is originally designed for monitoring the hydrophilic organic contaminants in wastewater (Alvarez et al., 2004) and is also increasingly recognised as a promising technique for the detection of pathogens in different matrices of water, offering a simpler and cost-effective alternative to conventional methods (Vincent-Hubert et al., 2021). Studies have demonstrated its effectiveness in detecting pathogens in both wastewater and surface water (Law et al., 2025). The use of low-cost materials, such as cotton gauze, to capture pathogens provides a practical and accessible option, particularly in RLCs (Haskell et al., 2024; Liu et al., 2023; Strike et al., 2025). Recent studies have explored a range of alternative passive sampling materials beyond cotton gauze, including granulated activated carbon (GAC), electronegative cotton-based swabs and tampons, polymeric resins and other sorbent matrices (Atoufi & Lampert, 2023; Hayes et al., 2022; Jones et al., 2022). These materials typically have a larger surface area and a greater affinity for nucleic acids (NA) or cells of bacteria, which enhances the effectiveness and reliability of passive sampling applications. Regardless of its advantages, passive sampling also has notable limitations. For instance, producing precise quantitative data from wastewater is a significant challenge because this technique provides a qualitative or semi-quantitative presence of pathogen rather than an estimate of load (Bivins et al., 2022). It also lacks standardised techniques for chemical analysis, data reporting and passive sampler applications, which makes it challenging to conduct comparisons across studies (Booij et al., 2016). These uncertainties can hinder the ability to figure out pathogen trends with clinical data, particularly in wastewater systems with a wide variety of contaminants.

### Sampling strategies

#### Hotspot-based sampling

Hotspot-based sampling strategy (HBSS) is a targeted surveillance approach used in areas where there are no sewage facilities available (Jakariya et al., 2021). Rather than relying on a comprehensive sewerage network, samples are collected from strategically identified sites, including wastewater drains, drainage sediments, canal water, and community septic tanks, prioritising rapid proxy on infectious agents and high-risk pathogen transmission (Amin et al., 2020; Jakariya et al., 2022). For example, hospital wastewater drains constitute highly complex effluents acting as a hotspot for antibiotic-resistant ESKAPE pathogens and ARGs (Rozman et al., 2020). Similarly, urban informal community open drains are commonly known hotspots for ARB transmission when there is insufficient sanitation infrastructure and frequent irrational use of antibiotics (Nadimpalli et al., 2020). Therefore, HBSS not only depends on sewage type but also on the context of infrastructural facilities and the pressure related to population behaviour for the spread of ARB and ARGs. For instance, in Kenya, the use of HBSS in informal settlements has proven effective, yielding 85% nonpathogenic *E. coli* and 15% pathogenic *E. coli* strains associated with diarrhoeal cases in Nairobi (Kiiru et al., 2024).

#### Decentralised sampling

Decentralised sampling strategy (DSS) is used in regions lacking centralised sewage systems. Sampling points can be established at locations such as community toilets, small drains, local sewers, septic tanks or sites of open defecation (Gonçalves et al., 2022). This approach can yield valuable data on pathogen prevalence in areas without direct access to large-scale sewage treatment facilities. Collection can be carried out through community-level initiatives (Danielsen et al., 2022). DSS offers a flexible and cost-effective method for pathogen monitoring in settings without central sewerage networks (Sha et al., 2024). For example, in Malawi, DSS has been implemented using in-pit latrines to monitor enteric pathogens (Capone et al., 2021). This strategy has demonstrated considerable potential for early outbreak detection and targeted intervention, particularly in RLCs (Gwenzi et al., 2023). Notably, the DSS sampling strategy emphasises the specific points of sample collection, with a focus on upstream or local sources, rather than the type of sample and the method of collection.

#### Alternative sampling

Alternative sampling strategy (ASS) targets areas with limited or no access to centralised sewerage systems (CSES). Potential sampling sites include local open drains, floodwater, washout pit latrines, septic tanks, wastewater from local businesses or informal settlements and public latrines or toilets (Okaali et al., 2022). This approach is particularly valuable in remote settings where conventional wastewater infrastructure is absent (O'Reilly et al., 2020). ASS, in contrast to DSS, relies on the sampling techniques where multiple samplers exist, but only a single sampler exists at a time based on sampling proportional to a given time interval, predefined conditions and variables, which enables monitoring shedding pathogens in regions where location-based sampling and autosamplers are not feasible.

### Detection of ESKAPE and resistance genes

The growing difficulty of detecting pathogens in wastewater has become one of the most significant challenges in RLCs due to a combination of technical and logistical constraints (Oon et al., 2023). The major factors, including limited trained personnel, cold-chain infrastructure, limited laboratory capacity and financial resources, often contribute to these constraints. Moreover, the higher cost and technical complexity of detection methods further challenge the sustainable detection of these pathogens and their associated genes. In recent years, labour-intensive scientific procedures have been transformed into more sophisticated approaches through the continuous advancement of analytical techniques (Hameed et al., 2018). Effective detection methods must strike a balance between sensitivity, cost, and technical complexity, while also being adapted to the specific context and needs of the target community (Váradi et al., 2017).

#### Culture-based techniques

Traditional culturing on selective media remains a reliable method for isolating ESKAPE pathogens. This approach encompasses several standard microbiological procedures used for their identification and characterisation. Often regarded as the “gold standard” and a conventional approach (Zhang et al., 2021), culture-based methods involve assessing colony morphology, performing Gram staining, examining morphological characteristics, conducting biochemical analyses and carrying out serotyping (Parasuraman et al., 2024). Antimicrobial susceptibility testing (AST), such as the Kirby–Bauer disc diffusion method, is employed to determine the response of ESKAPE pathogens to various antibiotics, thereby providing insight into resistance patterns and informing appropriate treatment strategies (Jawade et al., 2024; Pandey et al., 2021). Furthermore, automated platforms such as the Vitek 2 System enable rapid identification and AST of bacterial isolates, including ESKAPE pathogens, offering increased efficiency in clinical and surveillance contexts (Jawade et al., 2024).

#### Polymerase chain reaction (PCR)

Polymerase chain reaction (PCR) is a laboratory technique used to amplify specific DNA fragments through a simple enzymatic reaction involving a series of thermal cycles, enabling the detection of a specific gene (Jalali et al., 2017; Mehta, 2022). While conventional PCR can confirm the presence of DNA, it does not quantify the quantity of the DNA present. Quantitative PCR (qPCR), also known as real-time PCR, combines amplification with real-time detection, determining the quantity of the amplicon and analysing gene expression in a sample (Harshitha & Arunraj, 2021). Compared with conventional PCR, qPCR is faster, more efficient, highly specific and more sensitive, making it a preferred method for detection and quantification (Li et al., 2017). When appropriately designed, qPCR can achieve high specificity, sensitivity, and robustness of a PCR reaction, although the risk of false-positive results is dependent on SYBR Green dye–based assays and TaqMan Assays, primer and probe design (Cruz-Flores et al., 2019). For example, qPCR has been shown to detect as few as 8.42 × 10^1^ copies/µL, a sensitivity approximately 1000 times greater than that of conventional PCR, and it yields a higher positive detection rate (Sun et al., 2021). Although effective for qualitative detection, traditional PCR generally has a higher detection limit and lower sensitivity than qPCR. Conventional PCR does not provide information on the quantity of the target DNA. This is a significant limitation for applications requiring precise measurement of DNA concentration (McNair et al., 2025). qPCR, by contrast, can be applied to large numbers of samples to quantify pathogen DNA, a capability that is crucial for quantitative microbial risk assessment (Lopes et al., 2018). These characteristics make qPCR the superior choice for ESKAPE pathogen detection and quantification, particularly in clinical diagnostics and high-throughput surveillance settings.

#### Digital PCR and digital droplet PCR

Digital PCR (dPCR) and its variant, droplet digital PCR (ddPCR), are advanced molecular techniques that enable precise and highly sensitive detection of pathogens, including members of the ESKAPE group. Unlike traditional PCR or qPCR, both dPCR and ddPCR provide absolute quantification of nucleic acids (NA) without the need for standard curves, a feature that is essential for accurate pathogen detection (Chen et al., 2021a, 2021b; Lei et al., 2021). These methods are also less susceptible to PCR inhibitors, enhancing their reliability when analysing complex sample matrices (Maheshwari et al., 2017). ddPCR, in particular, is increasingly employed for the continuous monitoring of pathogen nucleic acids, a capability that is critical for effective infectious disease management and outbreak prevention (Yang et al., 2025).

#### Metagenomics

Metagenomics is a culture-independent, high-throughput sequencing approach that enables comprehensive analysis of all genetic material present in a sample. Several key methods are employed for the detection of ESKAPE pathogens using metagenomic techniques. These include next-generation sequencing (NGS), comprising short-read sequencing (e.g., Illumina) and long-read sequencing (e.g., Oxford Nanopore Technologies, ONT), as well as metagenomic next-generation sequencing (mNGS) (Raza et al., 2024). Hybrid assembly, which integrates both Illumina and ONT data, provides a more comprehensive characterisation by combining the strengths of each technology (Frias-De-Diego et al., 2025). mNGS can identify multiple pathogens without prior knowledge of the microbial community, making it particularly valuable for detecting unknown or rare pathogens. It can also deliver rapid nucleic acid (NA) insights from test samples (Han, 2022). In general, mNGS is a nontargeted, broad-spectrum pathogen screening technology that sequences all genetic material in a sample without the need for prior knowledge of the specific organisms present (Miller & Chiu, 2022; Simner et al., 2018).

#### Matrix-assisted laser desorption/ionisation time-of-flight mass spectrometry

Matrix-assisted laser desorption/ionisation time-of-flight mass spectrometry (MALDI-TOF MS) is a powerful proteomic technique that is widely used for the identification of microorganisms, including ESKAPE pathogens, and frequently outperforms traditional biochemical methods (Chen et al., 2021a, 2021b). The technique works by ionising proteins, primarily ribosomal proteins, from bacterial colonies and measuring their mass-to-charge ratios to produce a characteristic spectrum, which is then compared against a reference database (Liébana-Martos, 2018). MALDI-TOF MS is considered an economical option for microbial identification due to its speed, high reliability and the minimal cost of consumables per specimen (Calderaro & Chezzi, 2024).

#### Biosensors and nanotechnology

Biosensors and nanotechnologies are under active development for field-ready, portable detection devices. One notable application involves the integration of gold nanoparticles (gNPs) with specific antibodies or DNA probes for pathogen detection (Abu-Salah et al., 2015; Kulabhusan et al., 2022). Biosensors are analytical devices that incorporate biological elements, such as enzymes or antibodies, and typically detect targets through optical, electrical, thermal, or other measurable signals (Naresh & Lee, 2021). They can directly detect pathogens in untreated samples, thereby playing a significant role in quantifying pathogens in wastewater without the need for a separate sample preparation stage, which is essential for tracking public health status (Cui et al., 2021).

Nanosensors are another modern technique that promise the direct, rapid and precise detection and identification of various pathogens present in wastewater (Dimitrievska et al., 2023). A broad range of techniques is available, including enhanced oxidation processes, sorption, osmosis, ultrafiltration membranes, water remediation and disinfection using nanomaterials (Rafique et al., 2019). For real-time monitoring, nanosensors can be incorporated into lab-on-a-chip systems or portable devices for monitoring wastewater (Ghoorchian et al., 2022).

As shown in Table 1, while culture-based methods remain applicable in RLCs, approaches such as metagenomic and digital/digital droplet PCR (dPCR/ddPCR) are often preferred for their higher sensitivity. While each of the abovedescribed detection techniques has distinctive advantages, selecting a method for WBS in RLCs also requires considering the limitations of each method. Despite being cost-effective, culture-based methods are slow and inaccurately detect the presence of antibiotic resistance, given that they are unable to identify living but non-culturable bacteria. Although it is qualitative and needs constant electricity and a reagent supply, the conventional PCR method provides specificity. Quantitative PCR (qPCR) and digital PCR (dPCR) have higher sensitivity and quantification, but their everyday use is limited by the need for costly fluorescence systems, skilled personnel and thorough data processing. Metagenomics is extensively used for mapping resistomes, involves directly extracting and sequencing genomes of a group of microbes, and offers the most comprehensive technique. In contrast, it is still more resource-intensive, requiring advanced infrastructure, stable power supply, specialized expertise and substantially higher per-sample costs. ARB can be detected instantly using MALDI-TOF MS; however, it needs expensive equipment and a previous culture. Moreover, biosensors and isothermal assays still need to be validated despite their speed and portability. Therefore, while choosing methods for ESKAPE pathogen surveillance in RLCs, it is crucial to balance analytical accuracy, infrastructure, and cost.

**Table 1:** Summary of common detection methods for ESKAPE pathogens

Method	Targets	Advantages	Turnaround Time	Limitations	Applicability
Culture-based technique	Viable ESKAPE species	Low-cost, basic laboratory setup; confirms bacteria viability	24–72 h	Time-consuming, misses nonculturable organisms; labour-intensive and trained personnel	High (widely feasible in RLCs)
Conventional PCR	ARGs (e.g., mecA, blaNDM, vanA, and blaKPC)	Specific, relatively inexpensive and adaptable to field laboratories	6–8 h	Qualitative; requires reagents, a thermal cycler and trained personnel	Medium–high (adaptable with support)
qPCR	Quantification of ARGs in the wastewater samples	Sensitive, quantitative and relatively rapid	4–6 h	Requires fluorescence detection, a power supply and trained personnel	Medium (limited by access to equipment)
Digital PCR (dPCR/ddPCR)	High-precision ARG quantitation	Ultrasensitive and absolute quantification; low susceptibility to inhibition	4–6 h	High cost, limited availability and complex data analysis	Low–medium (pilot/research only)
Metagenomics	Entire resistome and taxonomic diversity	Comprehensive detection of unculturable organisms and novel ARGs	1–3 days	High cost; requires bioinformatics expertise and sequencing infrastructure	Low (research settings only)
MALDI-TOF MS	Rapid identification of species from cultured isolates	Fast and accurate species identification	< 1 h (postculture)	Requires prior culturing and expensive instrumentation	Low (select research hospitals)
Biosensors and isothermal assays	Specific ARGs (e.g., LAMP for blaNDM)	Portable, rapid and electricity-independent	< 30 min	Limited multiplexing, validation is needed	Medium to high (field-use potential)

## Wastewater-based surveillance of ESKAPE pathogens

**Fig. 1:**
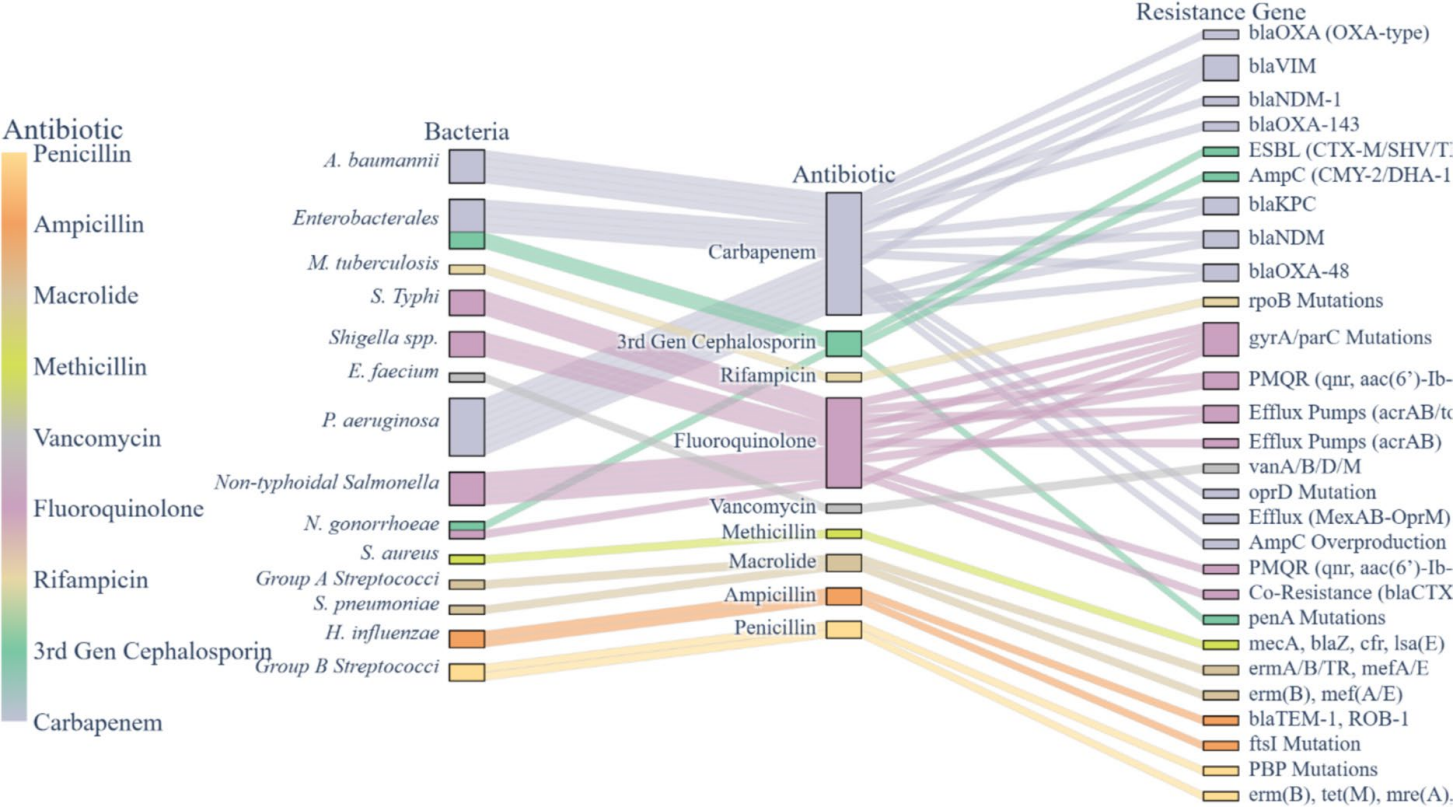
WHO Bacterial Priority Pathogens List (BPPL),  2024 update. The figure summarises the WHO’s 2024 update of the BPPL, highlighting antibiotics, ARB and associated ARGs. The bacterial pathogens are classified into priority levels based on their disease burden (mortality and morbidity), transmissibility, treatability and prevention options to inform global policy, research and development

The WHO’s updated bacterial priority pathogens highlights the critical threat posed by carbapenem-resistant and extended-spectrum beta-lactamase (ESBL)-producing bacteria, particularly in RLCs (Sati et al., 2025; WHO,^2024^). With high prevalence documented in East and West Africa, Latin America, and South Asia, the global spread of carbapenem-resistant and ESBL-producing bacteria, particularly among Enterobacterales, continue to be a major public health concern (Hays et al., 2022; Lincopan et al., 2023; Moges et al., 2021; Sampah et al., 2023). By connecting bacterial species to antibiotic classes and related mechanisms of resistance, including β-lactamases, efflux pumps, and target-site alterations, the 2024 update of the WHO list (Fig. 1) highlights the complexity of antibiotic resistance.

Given this, WBS provides an efficient and cost-effective method to monitor ARB and the spread of ESKAPE pathogens, particularly within RLCs where clinical surveillance is often limited. This section summarises the data on WBS detection of significant ESKAPE pathogens in Africa, Asia and Latin America, including genes and bacteria associated with resistance, patterns and public health surveillance implications.

### Methicillin-resistant S. aureus

WBS studies in several African and Asian countries have detected MRSA in both municipal and hospital wastewater, underscoring the potential of sewage-based monitoring for public health applications (Oladipo et al., 2019; Torabi & Rahimi 2021). The presence of the mecA gene, which confers methicillin resistance, is a key molecular marker frequently identified in wastewater samples using PCR-based techniques (Akya et al., 2020). For example, mecA was confirmed in 18 isolates collected from wastewater treatment plant (WWTP) effluents and surface waters in Central Brazil, with 15 of these isolates displaying multidrug-resistant phenotypes (Dos Santos et al., 2025). Similarly, Santos et al. (2020) reported that 36.7% of *S. aureus* isolates from drinking water sources in São Paulo carried the mecA gene. Another study in Kermanshah, Iran, found that 11% and 8% of isolates from raw and treated sewage, respectively, were MRSA, all of which harboured the mecA gene (Akya et al., 2020). Collectively, these findings highlight that the occurrence of MRSA in hospital wastewater and WWTPs poses a risk of environmental dissemination, underscoring the importance of ongoing environmental ARB surveillance (Silva et al., 2022).

The detection of vanA and vanB genes in sewage highlights the role of these facilities in contributing to environmental reservoirs of ARB (Farias et al., 2022; Furukawa et al., 2015). VRE have been identified at various stages of the WWTP process, suggesting that these systems may be unable to eliminate resistant bacteria and their associated genes (Oravcova et al., 2017). In support of this, Hamiwe et al. (2019) reported that WWTPs were ineffective in removing antimicrobial resistance genes (ARGs) from wastewater, as evidenced by the persistence of vanA, vanB and vanC genes in the final effluent, with the potential for their subsequent discharge into natural water bodies.

### Klebsiella pneumoniae and Enterobacter spp.

Research conducted in Tamil Nadu, India, identified *K. pneumoniae* and *E. coli* isolates with extended-spectrum β-lactamase (ESBL) properties in treated wastewater effluents (Boominathan et al., 2024). Similarly, Kagambèga et al. (2023) reported the dissemination of resistance genes, including bla_NDM_, bla_VIM_, bla_IMP_, bla_KPC_, and bla_OXA-48_ from carbapenemase-producing *E. coli* and *K. pneumoniae* in hospital effluents in Ouagadougou, Burkina Faso. In Brazil, studies have also identified multidrug-resistant (MDR) bacteria, including *K. pneumoniae*, in hospital wastewater, often carrying bla_TEM_ and bla_KPC_ genes associated with carbapenem resistance (Batista et al., 2023). High prevalence rates of ESBL-producing Enterobacteriaceae have been reported in various regions, with faecal carriage rates reaching 70.9% in rural Tanzania (Macha et al., 2025). In South Africa, *K. pneumoniae* has been found at high prevalence in rural hospital drains, suggesting its persistence throughout wastewater treatment processes (Mapipa et al., 2022). A study conducted in India and Mexico found that a significant proportion of *K. pneumoniae* isolates from wastewater are carbapenem-resistant, harbouring genes such as Furthermore, a study spanning India and Mexico revealed that a substantial proportion of *K. pneumoniae* isolates from wastewater were carbapenem-resistant, harbouring resistance genes such as bla_KPC_, bla_NDM_ and bla_OXA-48_ (Galarde-López et al., 2022; Sahoo et al., 2023a, 2023b).

### Acinetobacter baumannii

*A. baumannii* is frequently detected in hospital wastewater, urban sewage and river water, indicating substantial environmental contamination (Sahoo et al., 2023a, 2023b; Tsai et al., 2018). The continuous release of untreated or inadequately treated wastewater into the environment facilitates the dissemination of *A. baumannii*, thereby increasing the risk of community exposure (Music et al., 2017). In Nigeria, *A. baumannii* isolates have been found in both raw and treated hospital wastewater, with a high prevalence of carbapenem resistance genes such as bla_OXA-23_ and bla_NDM-1_ (Odih et al., 2023). Other OXA-type genes, including bla_OXA-51-like_, bla_OXA-65_, bla_OXA-66_, bla_OXA-23_, bla_OXA-51_, bla_OXA-72_,bla_OXA-23_, bla_TEM_ and bla_OXA-208-like_, as well as bla_OXA-117-like_, have also been identified in WWTPs. This diverse genetic repertoire contributes to the persistence and dissemination of carbapenem resistance in the environment (Higgins et al., 2018).

### Pseudomonas aeruginosa

The presence of multidrug-resistant *P. aeruginosa* (MDR-PA) in wastewater poses a significant public health threat due to the ongoing dissemination and proliferation of resistance genes, which complicates the treatment of infections caused by these strains (Okafor & Nwodo, 2023). Several studies have documented the occurrence of both MDR and XDR *P. aeruginosa* in wastewater. For example, 83% of isolates from hospital wastewater were resistant to at least one antibiotic from three or more classes, with 82% classified as MDR and 18% as (Miranda et al., 2015). In the same region, wastewater samples displayed high resistance rates to ceftazidime, cefepime, piperacillin–tazobactam, and imipenem, with isolates harbouring bla_NDM_ and bla_SPM_ genes (Monteagudo de Barros et al., 2024). Similarly, in South Africa, 55.6% of *P. aeruginosa* isolates from abattoir wastewater and aquatic environments were found to be MDR (Hosu et al., 2021). Furthermore, Okafor and Nwodo (Okafor & Nwodo, 2023) identified carbapenem resistance genes, including bla_IMP_, bla_KPC_ and bla_VIM_, in hospital wastewater from the Amathole District, South Africa. To mitigate the dissemination of these resistant strains, continuous surveillance is essential, alongside the implementation of improved wastewater treatment processes at primary source points before discharge into the wider sewerage network (Mahmud et al., 2025).

## Summary and research gaps

Detecting ESKAPE pathogens and life-threatening resistance genes in hospitals and wastewater is equally crucial. Evidence on how to reduce adverse health effects for communities in RLCs is necessary for effective policies and initiatives to eradicate ARB. However, the scope and geographic coverage of the study are still restricted, especially in Sub-Saharan Africa and some regions of South Asia. There is an urgent need to strengthen standardized WBS methodologies and assess the relationships between wastewater data and clinical antibiotic resistance developments to provide high-quality, evidence-based surveillance and incorporate WBS into national ARB surveillance systems in RLCs.

## Implementation of WBS in RLCs

The implementation of WBS in RLCs faces numerous challenges (Shrestha et al., 2021). There is a significant gap in published studies on the implementation of WBS for monitoring ESKAPE pathogens in most RLCs. This review paper indicates that the current studies on monitoring ESKAPE pathogens in RLCs are limited, within areas including sub-Saharan Africa and parts of Southeast Asia. Moreover, we assess existing knowledge about the disparities of different sample sources, sampling strategies and analytical methods that limit a comprehensive understanding of public health issues across regions. A key barrier is the inadequacy of wastewater infrastructure, as sewage networks in many RLCs are often underdeveloped, fragmented and hinder the collection of consistent and representative samples. This constraint is particularly critical in informal settlements and rural areas, where centralised sanitation systems are lacking (Aydamo et al., 2023). Technical and operational constraints also present significant barriers to the effective implementation of WBS. These challenges include insufficient laboratory infrastructure, limited access to advanced molecular tools, a shortage of trained personnel and restricted financial resources (Omohwovo, 2024). In addition, the absence of standardised protocols, inadequate data management systems and limited expertise in epidemiological modelling hinder the accurate analysis and interpretation of surveillance data (Cuadros et al., 2024). Integration with broader public health surveillance systems presents another significant challenge, often stemming from fragmented health infrastructure, weak intersectoral coordination and limited regulatory enforcement (Malakoane et al., 2020).

**Fig. 2:**
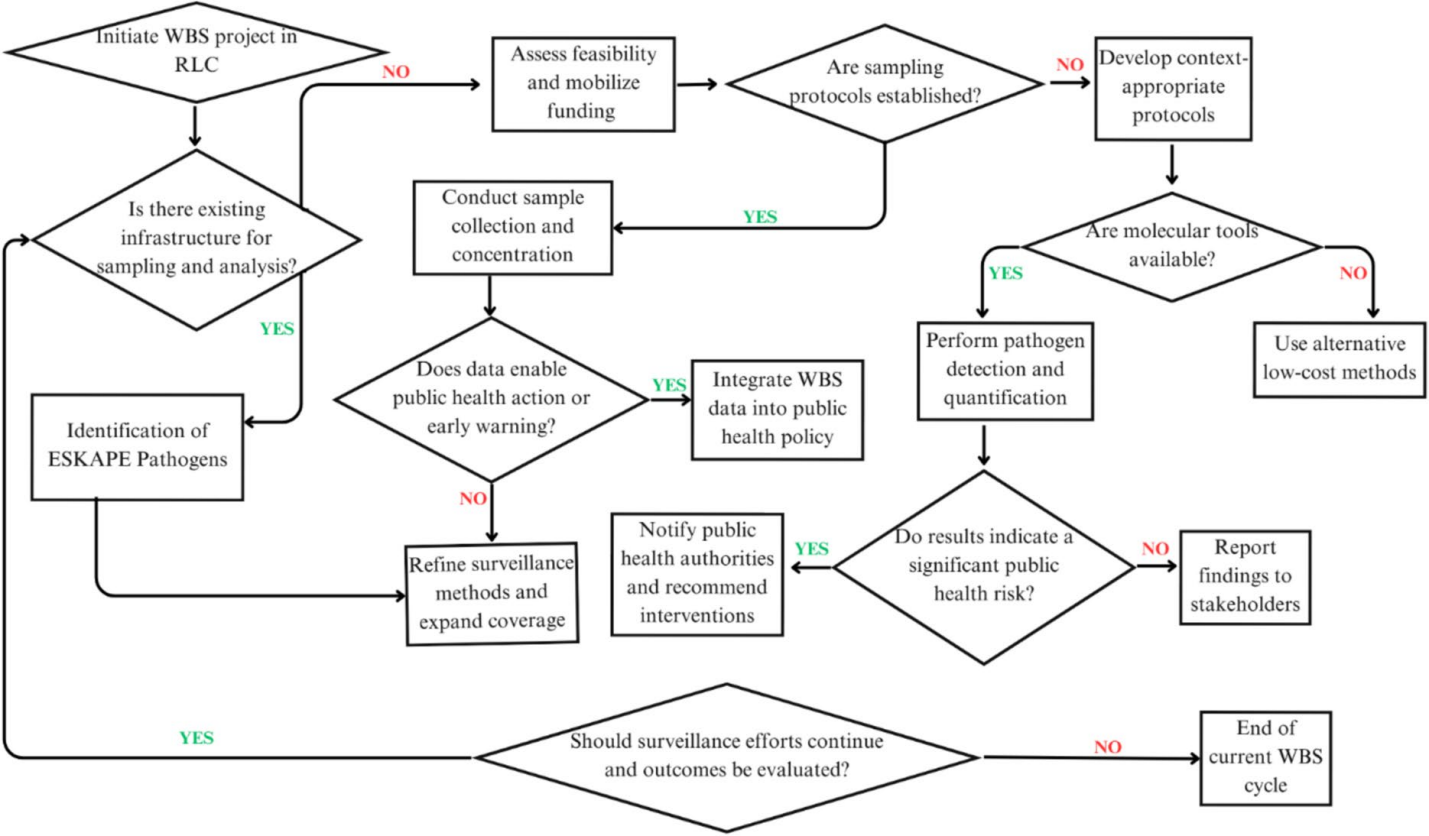
Conceptual framework for implementing the WBS in RLCs. This framework illustrates that a comprehensive WBS initiation starts with determining feasibility and infrastructure availability for data analysis. Furthermore, this framework emphasises the significance of linking WBS data with public health action to allow for early warning. Key recommendations derived from this framework include (**a**) establishing the ideal lab facilities, sampling strategies and personnel training to facilitate molecular tools for the detection of ESKAPE pathogens; (**b**) assessing a flexible WBS approach that combines cost-effective and advanced methods; (**c**) identifying the relative importance of interdisciplinary collaboration between environmental and healthcare authorities; and (**d**) assuring what future action to take concerning continuous surveillance for policymakers and researchers to allocate resources

To address the surveillance challenges, Fig. 2 presents a decision-making framework for implementing the WBS of ESKAPE pathogens in RLCs. This framework outlines a step-by-step process for determining feasibility, establishing sampling methods, carrying out detection, analysing findings and incorporating data into public health policy. This draws attention to a significant decision-making point where the choice of analytical techniques and the long-term sustainability of monitoring programmes are influenced by the availability of resources, surveillance instruments and trained personnel.

The case studies illustrated in Table 2 revealed targeted pathogens, surveillance strategies and challenges across regions in implementing the WBS approach to explore and understand ESKAPE pathogens that confer antibiotic resistance in RLCs. It is apparent from the findings in Africa and some parts of Asia that inadequate surveillance systems, insufficient wastewater infrastructure and weak collaboration with ARB programs are continuing challenges. Concerning the Latin American countries, such as Brazil and Mexico, the extant literature describes the same challenges, including inadequate regulation and poor wastewater management. Despite geographical variations, from the indicated table, this review confirms that the majority of studies rely on hospital and municipal wastewater sources. It assesses linkages between the extensive use of culture-based and molecular detection approaches, which promote the increasing initiative of surveillance, regardless of uneven distribution in relation to RLCs. It is worthwhile to clarify that these relationships indicate weaknesses and indicate the need for the regulated application of the WBS procedure, as it pertains to the achievement of enhanced ARB monitoring systems.

**Table 2:** A regional breakdown of key studies, targeted pathogens, surveillance strategies and WBS implementation challenges across RLCs

Region	Country	Targeted pathogen	Surveillance strategy	References	Notable challenges
Africa	South Africa	*Enterococcus* spp.,* K. pneumoniae*	Municipal WWTPs and hospital drains	Mapipa et al. (2022), Mbanga et al., 2021)	Inconsistent monitoring and fragmented surveillance
	Tanzania	*K. pneumoniae*, *P. aeruginosa*	Hospital wastewater	Karungamye et al. (2023)	Limited infrastructure and limited integration with AMR
	Ghana	*K. pneumoniae*	Pond effluents, hospital wastewater and domestic wastewater	Ekhosuehi et al. (2024)	Irregular waste disposal systems and limited integration with AMR
	Nigeria	*S. aureus, P. aeruginosa*	Abattoirs wastewater	Olawale et al. (2020)	Irregular waste disposal systems and inconsistent monitoring
	Ethiopia	*P. aeruginosa, S. aureus*	WWTP	Adugna and Sivalingam (2022)	Inappropriate wastewater treatment system and inconsistent monitoring
South Asia	India	*E. coli* and *K. pneumoniae*	Wastewater effluents	Boominathan et al. (2024)	Limited integration with AMR
	Bangladesh	*S. aureus*, *E. coli*, Enterobacter spp. and Klebsiella spp.	Hospital- and nonhospital-based wastewater treatment	Rahman et al. (2021)	Limited infrastructure and an inappropriate wastewater treatment system
Latin America	Brazil	Enterococcus, *S. aureus,* Enterobacteriaceae and *E. faecium*	Hospital sewage and Municipal WWTPs	Farias et al. (2022), Zagui et al. (2021)	Limited integration with AMR and a limited wastewater treatment system
	Mexico	*K. pneumoniae*, *A. baumannii*, *E. coli*, Citrobacter and Enterobacter	Hospital wastewater	Nolasco-Rojas et al. (2025)	Inadequate regulation and wastewater management practices
Southeast Asia	Thailand	*S. aureus*, *E. coli* and Acinetobacter spp.	Domestic and hospital WWTPs	Chiemchaisri et al. (2023)	Lack of effective wastewater treatment systems
	Vietnam	Enterobacteriaceae, Enterococcaceae, and Acinetobacter	Urban Wastewater	Vu et al. (2024)	Irregular monitoring
	Indonesia	*K. pneumonia*	Wastewater from the water tank	Simanjuntak et al. (2023)	Lack of effective wastewater treatment systems

## Practical considerations for implementing WBS in RLCs

### Cost-effective and low-maintenance solutions

The cost and complexity of WBS systems in RLCs can present a substantial barrier to implementation. Cost-effective, low-maintenance solutions reduce reliance on highly trained personnel, enabling local staff to undertake sampling, analysis, and maintenance with minimal external support. Such approaches also facilitate rapid troubleshooting, wider spatial coverage and long-term operational viability, thereby supporting the sustained surveillance of high-priority pathogens in wastewater (Gholipour et al., 2024; Shamsizadeh et al., 2024).

### Resilience to cold chain and power limitations

In many RLCs, such infrastructure is either unreliable or absent. Consequently, WBS tools that operate independently of cold chains and uninterrupted power, such as lyophilised reagents, solar-powered devices, and innovative point-of-care testing (POCT) technologies, represent critical advancements for pathogen detection in these settings (Leski et al., 2012; Li et al., 2023). These adaptations enable sensitive and accurate pathogen detection even in remote or off-grid environments, thereby significantly extending the reach and impact of surveillance activities.

### Tailored to local wastewater conditions

This aspect focuses on adaptability to challenging and variable environmental conditions in decentralised wastewater systems. WBS strategies should be tailored to address these constraints through, for example, rugged filtration systems, preconcentration techniques or flexible sampling protocols (Babler et al., 2023). Ensuring adaptability to local wastewater characteristics enhances the reliability of pathogen marker detection, thereby improving the accuracy and consistency of surveillance data.

### Incorporation of innovative, field-ready technologies

Innovative technologies are being increasingly incorporated into wastewater-based surveillance (WBS) systems to address operational challenges. For example, solar-powered sensors provide energy autonomy (Fan et al., 2021), smartphone-compatible readers enable rapid field analysis (Pawar et al., 2023), and dried reagent kits support sample testing without reliance on cold-chain storage (Lind et al., 2025), thereby facilitating on-site data collection and transmission. These tools help to overcome the logistical constraints of operating in resource-limited settings while enhancing real-time monitoring capabilities. Their integration supports a more agile, efficient and scalable pathogen surveillance, particularly in regions where traditional laboratory infrastructure is limited.

## Challenges and limitations in resource-limited countries

Limitations such as inadequate water treatment facilities, poor management, limited technical skills and the absence of real-time monitoring equipment further hinder frequent and accurate pathogen detection (Ateia et al., 2024; Cohen et al., 2024; Khuzwayo & Chirwa, 2020; Walsh et al., 2024). Additionally, WBS depends on robust laboratory infrastructure, but many RLCs face shortages in cold chain systems, biosafety labs and skilled technicians, often relying on donor-funded resources, which threatens the sustainability of surveillance programs. Creative solutions, like alternative sampling strategies piloted in South Africa, highlight efforts to overcome these barriers (James, 2021; Pocock et al., 2023).

Environmental and climatic factors also complicate WBS effectiveness, with conditions such as heavy rainfall, flooding, and drought impacting sample quality and pathogen detection (Talisuna et al., 2020). While WBS provides valuable community-level prevalence estimates, it cannot identify individual cases or precise locations (Hart & Halden, 2020). Ethical and privacy concerns further complicate its use, as monitoring public waste raises questions about transparency, oversight and community trust (Bowes et al., 2023; Nainani et al., 2024). Addressing these issues through clear communication and engagement is essential to responsibly advance WBS in RLCs.

## Strategic analysis of WBS implementation in RLCs

**Fig. 3:**
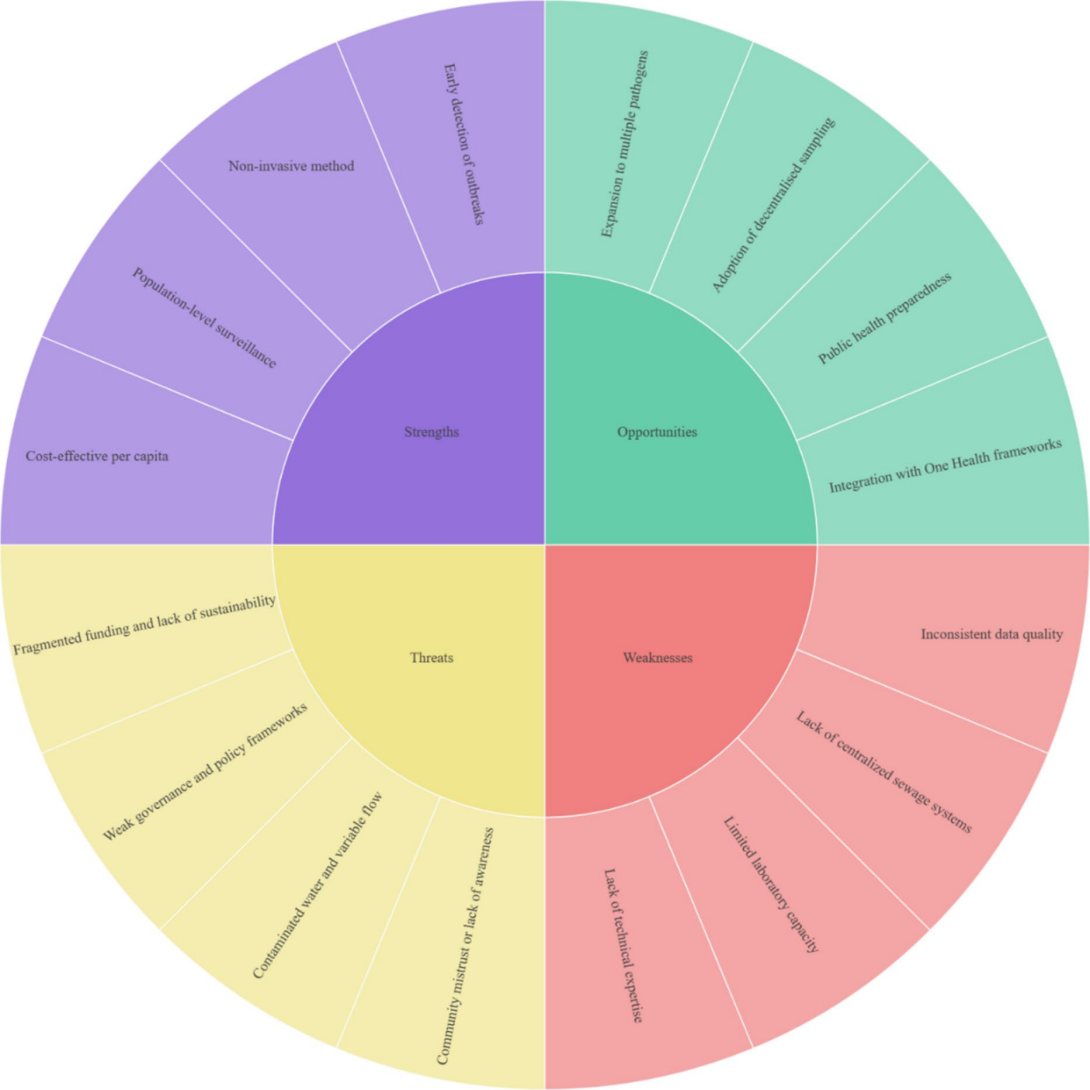
SWOT analysis of WBS in RLCs. This figure illustrates the strengths, weaknesses, opportunities and threats (SWOT) of implementing WBS for monitoring ESKAPE pathogens in RLCs and provides a basis for future investigations. This review is the first to evaluate the SWOT analysis of WBS in RLCs and provides new perspectives from different angles. Because of these characteristics, the practical strategy of adopting the WBS tool in RLCs for monitoring ESKAPE pathogens and antibiotic resistance is reliable. To the best of our knowledge, the strengths of WBS's current use in responding to antibiotic resistance are population-level surveillance, early detection, non-invasiveness, and cost-effectiveness, which promote more inclusive monitoring. The dependability and representativeness of the surveillance data are compromised by problems such as limited laboratory capacity, inconsistent data quality, insufficient centralised sewage infrastructure, and a lack of technical expertise. Therefore, addressing these factors is crucial for improving WBS effectiveness. Opportunities like decentralised sampling and wider pathogen coverage show potential for extending the role of WBS. Moreover, this review critically addresses fragmented funding, poor governance, contamination of water, and community mistrust as threats to the continued sustainability and effectiveness of WBS initiatives.

The strategic analysis of WBS implementation in RLCs highlights the balance between its potential benefits and existing challenges. The WBS detects disease outbreaks and monitors public health trends at the community level (Manirambona et al., 2024). This section presents a SWOT analysis of WBS in RLCs (Fig. 3).

## Conclusion

The emergence of antibiotic-resistant ESKAPE pathogens poses an important global concern. Wastewater offers valuable insights into the occurrence and potential spread of ARB, particularly in regions where surveillance systems are limited due to financial and infrastructural constraints. Wastewater analysis for antibiotic resistance complements conventional clinical surveillance as it reveals spatial and temporal patterns at a community level. WBS offers a practical, early detection method to track high-risk pathogens and support targeted public health interventions. It also offers a powerful, cost-effective and noninvasive approach for monitoring ESKAPE pathogens and resistance genes. Therefore, coordinated surveillance and control strategies are needed to overcome significant challenges related to infrastructure, laboratory capacity, data integration, and ethical governance for the successful implementation of WBS. Context-sensitive adaptations, such as decentralised sampling, low-tech detection tools and community participation, can facilitate successful implementation. This review also emphasises the urgency of investing in research in RLCs for cross-sector collaboration and the surveillance of ESKAPE pathogens by giving attention to the WHO bacterial priority pathogens list. By implementing a roadmap for integrating WBS into public health frameworks, stakeholders can leverage the WBS approach not only to monitor ESKAPE pathogens but also to inform proactive interventions, optimise resource allocation, and strengthen global efforts to combat antibiotic resistance. Generally, WBS is not merely a surveillance tool; it represents a scalable and cost-effective method to transform public health decision-making by aligning with international regulations, adopting shared strategies, and adhering to common wastewater quality standards. Future efforts must focus on scaling innovations, building capacity and embedding WBS in the core of public health decision-making by bridging critical surveillance gaps, particularly in RLCs.

## Data Availability

No original datasets were generated or analyzed for this review.
